# Sepsis neonatal tardía por SARS CoV-2

**DOI:** 10.7705/biomedica.5609

**Published:** 2020-11-12

**Authors:** Hernando Baquero, María Elena Venegas, Lorena Velandia, Fredy Neira, Edgar Navarro

**Affiliations:** 1 Centro de Investigación en Pediatría y Neonatología, Programa de Neonatología, Universidad del Norte, Barranquilla, Colombia Universidad del Norte Centro de Investigación en Pediatría y Neonatología Universidad del Norte Barranquilla Colombia; 2 Medicina de Alta Complejidad, S. A., MACSA, Hospital Niño Jesús, Barranquilla, Colombia Hospital Niño Jesús Barranquilla Colombia; 3 Programa de Neonatología, Universidad del Norte, Barranquilla, Colombia Universidad del Norte Universidad del Norte Barranquilla Colombia; 4 Departamento de Salud Pública, Universidad del Norte, Barranquilla, Colombia Universidad del Norte Universidad del Norte Barranquilla Colombia

**Keywords:** infecciones por coronavirus, recién nacido, sepsis neonatal, virus del SARS, Coronavirus infections, infant, newborn, newborn, neonatal sepsis, SARS virus

## Abstract

Durante la pandemia por SARS CoV-2 la gran mayoría de pacientes ha presentado afectación pulmonar como síntoma cardinal. En los niños, especialmente en recién nacidos, la sintomatología debida al efecto en otros sistemas diferentes al respiratorio puede dificultar el diagnóstico.

Se reportan tres casos de recién nacidos atendidos durante la fase de mitigación de la pandemia por SARS CoV-2 en el servicio de urgencias de un hospital materno-infantil en Barranquilla, Colombia, por presentar cuadros febriles que afectaban su estado general. En su evolución clínica predominó la sintomatología gastrointestinal sin que desarrollaran nunca manifestaciones respiratorias. La investigación epidemiológica no evidenció contacto con casos sospechosos o positivos para COVID-19. Sus madres no habían tenido síntomas respiratorios en los 45 días transcurridos desde la declaración de la emergencia en salud pública en el país. La ausencia de manifestaciones clínicas respiratorias en este grupo de pacientes con COVID-19 debe llamar la atención de los clínicos sobre la necesidad de sospechar la infección por SARS CoV-2 en recién nacidos con estados febriles.

## Abstract (en)

During the SARS COV-2 pandemic, the vast majority of infected patients are showing symptoms related to lung damage. At pediatric ages, especially newborns, symptoms from other organ systems without respiratory illness could make COVID-19 hard to diagnose. We are reporting three cases of newborns who were attended in the course of the mitigation phase in the emergency service of a maternal hospital in Barranquilla, Colombia, for high temperature and general compromised condition. During their clinical course, they developed gastrointestinal symptoms without showing any respiratory manifestations. They were not epidemiologically linked to a contact suspected to be a COVID-19 case and their mothers had had no respiratory symptoms since the public health emergency in our country was declared 45 days before. The absence of clinical respiratory manifestations in this group of patients with COVID-19 should draw clinicians' attention to the need to suspect SARS CoV-2 infection in febrile newborns.

El coronavirus (SARS CoV-2), agente etiológico de la actual infección pandémica, recibe su nombre del inglés *severe acute respiratory syndrome coronavirus.* Este virus ARN pertenece a la familia Coronaviridae, cuyos miembros producen en los humanos infecciones respiratorias con fiebre, tos y disnea y, ocasionalmente, manifestaciones gastrointestinales ^1^.

Hasta la fecha no se ha documentado la infección vertical por SARS CoV-2. Por la edad de aparición de las manifestaciones clínicas y la imposibilidad de documentar la presencia viral en el líquido amniótico y el tejido placentario, las escasas series de casos publicados de COVID-19 neonatal sugieren que la trasmisión es horizontal, por contagio con casos positivos para la enfermedad ^2^^-^^4^.

En el estudio retrospectivo de Wei, *et al.*^5^, se describen nueve pacientes menores de un año de edad diagnosticados en China con COVID-19, pero ninguno de ellos era menor de un mes. El curso de la enfermedad fue benigno en todos ellos. El reporte oficial de casos del Instituto Nacional de Salud de Colombia no determina cuáles corresponden a recién nacidos.

Hasta el 19 de mayo del 2020 se habían reportado 96 casos en menores de un año, tres de los cuales fallecieron y nueve fueron atendidos en unidades de cuidados intensivos ^6^.

Se presenta el reporte de tres casos de recién nacidos diagnosticados con COVID-19, cuyas madres no habían manifestado síntomas compatibles con la enfermedad.

## Caso 1

Se trata de un recién nacido de 26 días de vida, procedente del área metropolitana de Barranquilla, de raza mestiza, producto del segundo embarazo y nacido por cesárea debido a bradicardia fetal; su peso al nacer fue de 3.100 g, la edad gestacional era de 38 semanas. Fue llevado a urgencias por presentar fiebre de tres días de evolución. La madre tenía 20 años de edad y era migrante irregular extranjera con pareja estable. La vacunación durante la gestación había sido adecuada y las serologías para HIV, hepatitis B, toxoplasmosis y sífilis (VDRL) no evidenciaron infección activa. Refirió que vivía en condiciones de hacinamiento y negó haber tenido contacto con personas que presentaran síntomas respiratorios en el último mes.

En el momento del ingreso, el niño se mostraba activo y reactivo, afebril, con los signos vitales en los límites normales; no se registraron datos sobre enfermedades en el examen físico. La impresión diagnóstica determinó la presencia de sepsis neonatal tardía, por lo que se hizo un estudio séptico y se inició el tratamiento con ampicilina y amikacina en las dosis usuales. Durante el primer día de hospitalización el estado general del niño se deterioró, con distensión abdominal acentuada, mala perfusión periférica y sangrado por sonda orogástrica.

En los exámenes de laboratorio se encontró hemoconcentración, leucopenia, función hepática alterada y dímero-D elevado, en tanto que la proteína C reactiva (PCR) fue negativa. Dado el riesgo epidemiológico por el lugar de residencia de la madre, se solicitó una PCR-RT para SARS CoV-2 en el segundo día de hospitalización, la cual resultó positiva (muestra de hisopado de nasofaringe). Sus síntomas se manejaron con hidratación endovenosa y tratamiento de sostén. La evolución fue satisfactoria y se le dio egreso en el décimo día de tratamiento (cuadro 1).

**Cuadro 1:** Valores registrados en el momento del ingreso de los pacientes

	Caso 1	Caso 2	Caso 3
Edad gestacional (semanas)	38	39	38
Días de vida al ingreso	26	16	20
Hemoglobina (g/dl)	19,6	15	14
Hematocrito (%)	58,7	45,2	42
Leucocitos (por mm^3^)	10.800	11.000	11.500
Plaquetas (por mm^3^)	383.000	224.000	105.000
Glucemia (md/dl)	115	133	
Bilirrubina indirecta (mg %)	0,6	9,8	1,53
Bilirrubina directa (mg %)	1,12	1,15	3,3
Dímero D (<200 ng/ml)	1.000	726	491
LDH (valor normal: 230-460 U)	1.227	777	-
Fibrinógeno (valor normal: 200-393 mg/dl)	-	98	274
AST (valor normal: 5-34 U/L)	18	74	88
GPT (valor normal: 0-55 U/L)	138	71	64
PCR	Negativa	Negativa	Negativa

LDH: lactato deshidrogenasa; AST: aspartato aminotransferasa; GPT: transaminasa glutámico pirúvica

Las pruebas de PCR-RT en las muestras de hisopado nasofaríngeo de su padre y su madre se reportaron como positivas y se los clasificó como casos.

## Caso 2

Se trata de un recién nacido de 16 días de vida, procedente del área metropolitana de Barranquilla, afrodescendiente, producto del cuarto embarazo y nacido por cesárea motivada por una anterior. Su peso al nacer había sido de 3.600 g con una edad gestacional de 39 semanas; fue llevado a urgencias por presentar un cuadro de tres días de evolución con deposiciones líquidas acompañadas de sangre fresca en las últimas 24 horas. La madre tenía 39 años de edad, había recibido la vacunación adecuada durante la gestación y la serología para HIV, hepatitis B, toxoplasmosis y VDRL no evidenció una infección activa. Había sido hospitalizada en cuatro oportunidades por infección recurrente de vías urinarias y litiasis renal. El cultivo rectovaginal fue positivo para estreptococo del grupo B y negó haber tenido contacto con personas que presentaran síntomas respiratorios en el mes anterior.

En el ingreso, el niño presentaba apariencia tóxica y estaba ictérico, por lo que la impresión diagnóstica fue de sepsis neonatal tardía y sospecha de enterocolitis necrosante (estado 1B) y trastorno hemorrágico del recién nacido. Se solicitó una PCR-RT para SARS CoV-2 cuyo resultado fue positivo (muestra de hisopado de nasofaringe). Se inició el tratamiento con ampicilina y amikacina en las dosis usuales.

En los exámenes de laboratorio se encontró que la función hepática estaba alterada y que el dímero D, el fibrinógeno y la deshidrogenasa láctica estaban elevados; los tiempos de coagulación eran prolongados, en tanto que la PCR fue negativa. El urocultivo fue positivo al tercer día de hospitalización *(Eschericha coli* sensible) y el paciente evolucionó satisfactoriamente. Se le dio egreso al séptimo día de tratamiento (cuadro 1).

La prueba de PCR-RT en la muestra de hisopado nasofaríngeo de su madre se reportó como positiva y se la clasificó como un caso asintomático.

## Caso 3

Se trata de un recién nacido de 20 días, procedente de Barranquilla, mestizo, producto del primer embarazo y nacido por cesárea motivada por dilatación estacionaria. Su peso al nacer fue de 3.750 g y su edad gestacional de 38 semanas; fue llevado a urgencias por un cuadro de cuatro días de evolución con intolerancia a la vía oral, lesiones pustulosas en la piel del cuello e ictericia. La madre tenía 18 años de edad, había recibido la vacunación gestacional adecuada y la serología para HIV, hepatitis B, toxoplasmosis y VDRL no evidenció ninguna infección activa. Negó haber tenido contacto con personas con síntomas respiratorios en el mes anterior.

Al ingreso, el paciente tenía apariencia tóxica, estaba ictérico y afebril; la impresión diagnóstica se determinó como de sepsis neonatal tardía y sospecha de enterocolitis necrosante (estado 1). Se solicitó la PCR-RT para SARS CoV-2, la cual fue positiva (muestra de hisopado de nasofaringe). Se inició el tratamiento con ampicilina y amikacina en las dosis usuales.

En los exámenes de laboratorio se encontró anemia, trombocitopenia, dímero D elevado y función hepática alterada, en tanto que la PCR fue negativa. El paciente evolucionó satisfactoriamente y se le dio egreso al séptimo día de tratamiento (cuadro 1).

Las pruebas de PCR-RT en muestras de hisopado nasofaringeo tomadas a su padre, su madre y a los contactos domiciliarios se reportaron como negativas.

En este grupo de recién nacidos la proteína C reactiva (reactante de fase aguda con un alto valor predictivo de la inflamación) era negativa en el momento del ingreso y durante el curso de la hospitalización, lo que sugiere que la inflamación es poco frecuente en la evolución de la enfermedad (cuadro 1).

## Consideraciones éticas

Este reporte de caso fue aprobado por el Comité de Ética en Investigación del Área de la Salud de la Universidad del Norte.

## Discusión

Cuando se produce una infección viral durante el embarazo, su transmisión intrauterina suele generar complicaciones serias en los niños. El último agente infeccioso incluido en el grupo de infecciones perinatales por contagio vertical fue el virus del Zika ^7^^,^^8^. Aunque hasta el momento ha habido, por lo menos, un reporte de la presencia del SARS CoV-2 en las células del sincitiotrofoblasto de la placenta de una mujer embarazada sintomática para COVID-19 con preeclampsia grave y desprendimiento de la placenta ^9^, aún no hay evidencia de transmisión vertical en madres con infección activa sintomática en el momento del parto ^10^^,^^11^.

En una serie de 214 mujeres gestantes a quienes se les hizo tamización universal con PCR-RT en muestras de cepillado nasofaríngeo para detectar SARS CoV-2 en el momento del parto, se encontró una positividad del 15,4 % (33/214), aunque solo cuatro tenían síntomas de la enfermedad en el momento de la hospitalización ^12^. Este tipo de pruebas ayudaría a detectar madres asintomáticas para así disminuir las posibilidades de contagiar al recién nacido en el periodo neonatal con medidas de aislamiento respiratorio, distanciamiento físico e higiene frecuente y adecuada de las manos. Tales medidas podrían contribuir a salvaguardar la salud de los neonatos, pues todavía no se ha documentado la presencia del SARS CoV-2 en la leche materna ^10^.

La enzima convertidora de angiotensina 2 (ACE2), una metalopeptidasa, se ha identificado como el receptor funcional del SARS CoV-2. Se sabe que el ARNm de la ACE2 está presente en muchos órganos, pero su expresión proteica no se conoce del todo. Hamming, *et al.*^13^, reportaron su expresión superficial en células epiteliales alveolares pulmonares y en enterocitos del intestino delgado. También fue posible identificarla en las células endoteliales arteriales y venosas y en las del músculo liso arterial de varios órganos, lo que ha contribuido al conocimiento de la patogenia de las principales manifestaciones clínicas de la COVID-19.

El tropismo del SARS CoV-2 por el tracto gastrointestinal, así como su detección en materia fecal ^14^^,^^15^ pueden explicar perfectamente los cuadros gastrointestinales reportados en varias series de pacientes ^16^, una de las cuales incluía diez niños con síntomas inespecíficos y escaso compromiso pulmonar. En ocho de ellos la prueba de PCR-RT en las muestras de escobillado rectal seguía siendo positiva aún después del resultado negativo de las pruebas realizadas en las muestras de nasofaringe, lo que aumentaría la posibilidad de la transmisión fecal-oral ^17^.

En los pocos casos publicados de COVID-19 en recién nacidos se ha podido documentar que el curso de la enfermedad es más benigno. La sintomatología en ellos suele manifestarse con una sepsis tardía; llama la atención que la afectación pulmonar no se ha descrito tan frecuentemente como en los grupos de mayor edad ^18^. La interacción del SARS CoV-2 con el sistema inmunitario de los adultos, y su posterior respuesta disfuncional, marca la progresión de la enfermedad ^19^. Por su inmadurez inmunológica, el curso y la gravedad de la enfermedad en los recién nacidos podría ser completamente diferente.

### Implicaciones para la clínica

Los mecanismos fisiopatológicos de la COVID-19 incluyen la infección y el compromiso del tracto gastrointestinal. En los recién nacidos con manifestaciones gastrointestinales, febriles o afebriles, evaluados por cuadros sugestivos de sepsis tardía durante la pandemia debe hacerse la prueba de detección de la infección por SARS CoV-2, independientemente de que haya o no un nexo epidemiológico demostrado. La presencia de material viral en la materia fecal de los pacientes afectados debe motivar medidas de bioseguridad específicas para este tipo de contaminación.
